# Effects of salicylic acid, benzothiadiazole, and other commercial biostimulants on boosting faba bean (*Vicia faba* L.) tolerance against *Orobanche* spp

**DOI:** 10.1371/journal.pone.0324976

**Published:** 2025-06-09

**Authors:** Amal Bouallegue, Faouzi Horchani, Siwar Thebti, Imen Trabelsi, Zayneb Kthiri, Moez Amri, Mohamed Kharrat, Zouhaier Abbes

**Affiliations:** 1 Field Crop Laboratory, National Institute for Agricultural Research of Tunisia (INRAT), University of Carthage, Tunis, Tunisia; 2 Laboratory of Biotechnology and Biomonitoring of the Environment and Oasis Ecosystems (LBBEEO), Faculty of Sciences of Gafsa, University of Gafsa, Gafsa, Tunisia; 3 Agronomic Sciences and Techniques Laboratory, National Institute for Agricultural Research of Tunisia (INRAT), University of Carthage, Tunis, Tunisia; 4 Genetics and Plant Breeding Laboratory, National Institute of Agronomy of Tunis, Tunis, Tunisia; 5 AgroBioScience Program (AgBS), College of Sustainable Agriculture and Environmental Science (SAES College, Mohammed VI Polytechnic University (UM6P)), Ben Guerir, Morocco; Abdul Wali Khan University Mardan, PAKISTAN

## Abstract

Broomrapes (*Orobanche* spp.) caused important agricultural problems reducing faba bean cultivated area and production in Tunisia. The effect of chemical tolerance inducers (Salicylic Acid SA and Benzothiadiazole BTH) and commercial biological tolerance inducers (Serenade, Trianum-P and Panoramix) on the induction of tolerance to *O. foetida* and *O. crenata* on faba bean was studied under field and controlled conditions experiments. These tolerance inducers were evaluated on the susceptible small seeded faba bean cv. Bachaar and applied as seeds’ pretreatment (seeds priming – coating). SA and BTH proved to be the best seed pretreatment inducers that reduced *O. foetida* infestation and increased plant growth and seed yield under field and controlled conditions. Induced tolerance was associated with reduced orobanche seed germination rate and tubercle number. Biological tolerance inducers reduced orobanche infestation under controlled conditions especially in quadratic plastic dishes but not under field conditions. In absence of complete resistance to broomrape, these results support the evidence of using tolerance inducers to control broomrapes and that could be considered as one additional component in an integrated control strategy.

## Introduction

Faba bean (*Vicia faba* L.) is among the most cultivated grain legumes in the Mediterranean region and China. It plays important agronomic and socio-economic roles. In Tunisia, faba bean represents the most important legume crop with 72% of total grain legume cultivated area and average areas of 53,820 ha from 2018–2022 [1]. However, production, yield and growing areas are variable from year to year, especially due to climatic variation, diseases and pests. Infestation by broomrapes is considered one of the most important factors reducing faba bean yields in Tunisia [2–5]. Broomrapes (*Orobanche* spp. and *Phelipanche* spp.) are obligate parasitic plants lacking chlorophyll and unable to synthesize their own assimilates. Broomrapes infestation is located mainly in the Mediterranean region and the Middle East, but can also be found in a similar climate in Australia and California [6]. They are entirely dependent on their hosts for their nutritional requirements. Broomrapes cause important damage in many crops worldwide. In Tunisia, two Orobanche species (*O. foetida* Poiret and *O. crenata* Forsk.) cause important yield losses that can reach more than 90% in faba bean highly infested fields [7,8]. Several control strategies were tested [8–11], but none of them have resulted in a complete and successful control of the parasite.

Several studies showed that the use of tolerance inducers enhanced natural defenses of the plant to control pathogens, leading to a systemic acquired resistance (SAR) [12–15]. SAR has been identified as an effective tool for controlling fungi, viruses, bacteria, and parasitic plants [16,17]. It can be induced by the application of chemical tolerance inducers such as salicylic acid (SA) or benzo (1,2,5) thiadiazole – 7 – carbothioic acid S-methyl ester (BTH) [18,19] or by products of biological origin called biostimulants [20–23].

SA is considered as hormone-like substances involved in the regulation of several physiological processes and induce protection against abiotic and biotic stresses in plants [24–28]. It is considered as a signal molecule that plays an important role in the induction of SAR in plants through the activation of defense compounds including phenolic acids, pathogenesis related proteins and flavonoids [29,30]. Several studies on different plant species such as clover [31], faba bean [19,32,33], pea [18], lentil [34], tobacco, hemp [35], sunflower [36–38] and rapeseed [39] have indicated that BTH and SA applications have induced SAR against broomrapes. Biostimulants are reported to have a positive effect on crop yield by stimulating plant development and improving plant resistance to biotic and abiotic stress [20,22,40–42]. Previous studies have reported that the use of biostimulants can activate resistance to pathogens by regulating hormonal balance, improving photosynthetic capacity and nutritional absorption and efficiency [43–45]. The use of biostimulants in agriculture can help achieving sustainable crop production with minimal environmental negative impact. These biostimulants might include inorganic or organic materials, chemical elements and also beneficial microorganisms. Recently, some commercial biostimulant products include several beneficial microorganisms such as *Bacillus* spp., *Trichoderma* spp., and arbuscular mycorrhizal fungi [22]. Peng et al. [46] reported that Serenade (a fungicide that includes *Bacillus* spp.) reduced the damage caused by different fungal diseases in many crops. Similarly, other studies reported the positive effect of Trianum-P (a *T. harzianum*-T22-based biofungicide) promoted plant growth and plant diseases control [47,48]. It was also reported that Panoramix (a product that includes *Bacillus* spp., *Trichoderma* spp., arbuscular mycorrhizal fungi and polysaccharides) had a significant effect on promoting plant growth [49].

Chemical and biological tolerance inducers are generally applied to the plant by seed pretreatment, seed coating, foliar spray or root drench and/or by direct application to the soil [22,26]. Among these methods, seed coating and priming are the most effective techniques because they allow the use of low quantities of microorganism solutions in a specific application. There is little documented literature on the effects of exogenous biostimulants on *O. foetida* infestation in faba bean. The aim of this study was to test the effect of chemical and biological tolerance inducers (biostimulants) on the response of faba bean to broomrape infestation in the field and under controlled conditions.

## Materials and methods

### Plant material

The small seeded faba bean cv. Bachaar, used in this study, was selected and developed by INRAT – Tunisia for its high productivity in Orobanche-free fields and tolerance to local rust races (*Uromyces viciae-fabae*) and stem nematode (*Ditylenchus dipsaci*). This variety was reported to be susceptible to *O. foetida* and *O. crenata* [7,8]. Faba bean seeds were provided by Field Crops Laboratory – National Institute of Agricultural Research INRAT- Tunisia. *O. foetida* and *O. crenata* seeds were collected during the cropping season 2019–2020 from mature orobanche shoots parasitizing faba bean plants, respectively, from Beja and Ariana (Tunisia).

### Seed pretreatment and coating

After sterilization with 5% calcium hypochlorite and rinsing with sterile distilled water, faba bean seeds were (i) soaked in distilled water or (ii) pretreated with salicylic acid SA (0.1 or 1 mM) or Benzothiadiazole BTH (0.05 g/L) or (iii) coated with Serenade, Trianum-P or Panoramix (Koppert Biological Systems, Rotterdam, The Netherlands) (Table 1). The coating technique consisted of mixing the coating product with water. Then, the coating mixture was gradually applied to the faba bean seeds in continuous rotation, until complete adhesion, absorption and homogeneous distribution among the seeds.

**Table 1:** Different treatments and doses applied in field and controlled conditions.

Treatment	Composition	Dose
Control	Water	–
SA1	Salicylic acid	0.1 mM
SA2	Salicylic acid	1 mM
BTH	(1,2,5) thiadiazole – 7 – carbothioic acid S-methyl ester	0.05 g/L
Trianum	*Trichoderma*	0.5 g/L
Serenade	*Bacillus* spp.	8 mL/Kg
Panoramix	*Bacillus*, *Trichoderma*, Myccorhize, Polysaccharides	8 mL/Kg

### Quadratic plastic dish experiment

The quadratic plastic dishes (120 x 120 x 17 mm, Greiner) were filled with sterilized sand, moistened with water and then covered with a water-imbibed fiber glass filter paper. Three perforations were made in plastic dishes; one to allow the stem out of the dish and the two others for root feeding in culture medium. Sterilized Orobanche seeds (15 mg = approximately 7,500 orobanche seeds per dish) were spread on the filter paper. Plastic dishes were closed and vertically stored in a plastic basin and placed in darkness at 21°C for 10 days and watered as necessary. Pre-germinated sterilized faba bean seeds were placed on the fiber glass filter paper in contact with the preconditioned orobanche seeds in the dishes. Seven treatment groups were prepared including the control and the treatments using the different plant tolerance inducers (Table 1). Eight replicates for each treatment were done. Plastic dishes were kept in the glasshouse at 25 ± 3°C and 70% humidity. Thirty-five days after inoculation (DAI), *Orobanche* spp. seed germination percentage was evaluated by using a binocular microscope. Tubercle formation was also counted one month later.

### Pot experiment

Faba bean plants were grown in 2 liters’ pots filled with sterilized soil. Orobanche pots inoculation consists in adding 20 mg of *O. foetida* or *O. crenata* seeds per Kg of soil (approximately 12,500 orobanche seeds per pot). Non inoculated pots served as control. Pots were subdivided into seven groups (Table 1). All pots (one faba bean seedling per pot) were arranged in randomized complete block design with five replications and watered as needed. The plants were grown under glasshouse conditions at 20 ± 3 °C, 70% humidity and a 16 h photoperiod.

At pod setting stage (four months after sowing), faba bean roots were removed from the soil, gently washed, and the number and the dry weight (DW) of *O. foetida* and *O. crenata* infestation per plant and the host shoot dry weight per faba bean plan were determined.

### Field evaluation trial

The trial was conducted under high *O. foetida* infested plot at Oued Beja research station – Tunisia (36°43’N, 9°12’E), during the two cropping seasons 2020–2021 and 2021–2022 and according to a randomized complete block design (RCBD) with three replications. Faba bean seeds were sown in four rows of 4 m length and 0.5 m inter-row spacing. Sowing was done during the last week of November. The same faba bean seed treatments as field and pot experiments were applied (Table 1).

The trial was not subjected to fertilizers’ application, only hand weeding was done when required. At crop maturity, infestation and agronomic parameters such as *O. foetida* number, incidence, severity [7], seed yield per plant and 100 seeds’ weight were recorded.

### Statistical analysis

The statistical analyses were conducted using SPSS software (Version 23.0 for Windows). ANOVA was performed employing a general linear model, with treatments considered as fixed factors. All measurements were carried out at least in triplicate. Significance levels were set at P = 0.05 and Duncan’s multiple-range test was employed for pairwise comparisons.

## Results

### Quadratic plastic dish experiment

**Fig 1:**
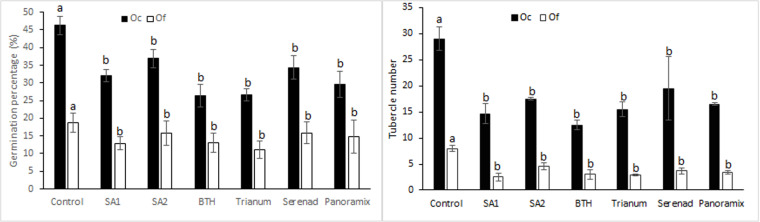
Effects of chemical and biological tolerance inducers on *O. foetida* and *O. crenata* seeds’ germination (a) and tubercles’ number (b) in faba bean assessed in Petri dish assay. Data are means ± Standard Error (SE). For each Orobanche specie, data with the same letter are not significantly different (Duncan, P = 0.05).

The used chemical and biological tolerance inducers significantly reduced the germination percentage of both *O. foetida* and *O. crenata* seeds (Fig 1). Decreases ranged from 25.84 to 45.72% for *O. foetida* and from 20.22 to 42.94% for *O. crenata*. BTH treatment showed the highest decrease of Orobanche seed germination whereas the lowest decreases were recorded with Serenade and AS2 treatments (Fig 1). All used tolerance inducers reduced *O. foetida* tubercles number by 41.62 to 68.75% and 32.76 to 56.90%, respectively for *O. foetida* and *O. crenata*. AS1 and BTH treatments manifested the highest decreases while AS2 and Serenade treatments showed the lowest decreases (Fig 1), respectively for *O. foetida* and *O. crenata*. Attached parasites did not show necrosis, regardless of the treatment used.

### Pot experiment

**Fig 2:**
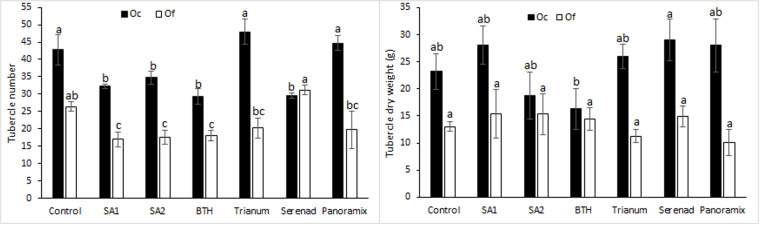
Effects of chemical and biological tolerance inducers on *O. foetida* and *O. crenata* tubercles’ number (a) and tubercles’ dry weight (b) in faba bean assessed in pot assay. Data are means ± Standard Error (SE). For each Orobanche species, data with the same letter are not significantly different (Duncan, P = 0.05).

ANOVA showed significant differences between the seed coating treatments for Orobanche shoots number and faba bean shoot dry weights. No significant effect was observed for Orobanche dry weight (Fig 2). SA1, SA2 and BTH seed treatments resulted in significant decreases of *O. foetida* tubercles’ number with 35.61, 33.71 and 31.82%, respectively. However, decreases of 31.66, 30.84, 26.65 and 18.69% were recorded for *O. crenata* tubercles’ number in response to BTH, Serenade, SA1 and SA2 seeds treatments, respectively (Fig 2). Compared to the control treatment, SA and BTH treatments revealed the maximum decreases of the tubercle number for both orobanche species. No tubercle necrosis was observed for all the tested treatment.

**Fig 3:**
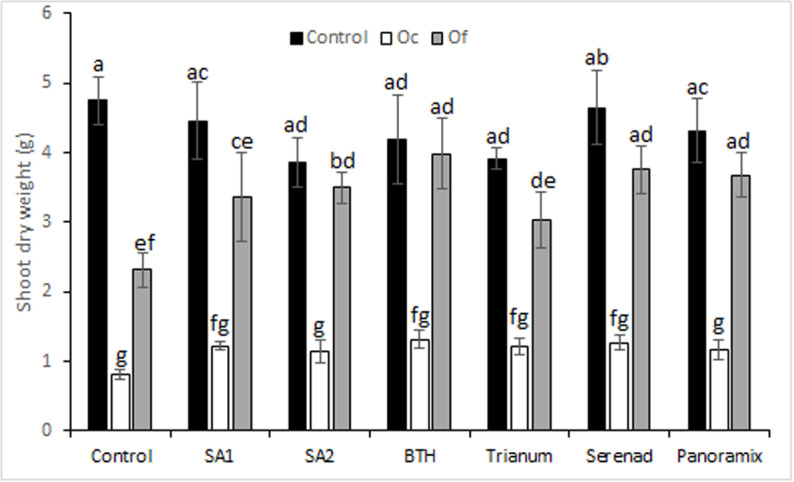
Effects of chemical and biological tolerance inducers on faba bean shoot dry weight in faba bean assessed in pot assay. Data are means ± Standard Error (SE). Data with the same letter are not significantly different (Duncan, P = 0.05).

Both Orobanche species showed significant decrease in shoot DW of untreated plants compared to control. After application of elicitor treatments, no significant differences were recorded for shoot dry weights except a slight increase in shoots compared to infested untreated plants (Fig 3). This increase ranged from 24.59 to 50.61% for *O. foetida* infested plants and from 6.45 to 47.95% for *O. crenata* infested plants.

### Field experiment

**Fig 4:**
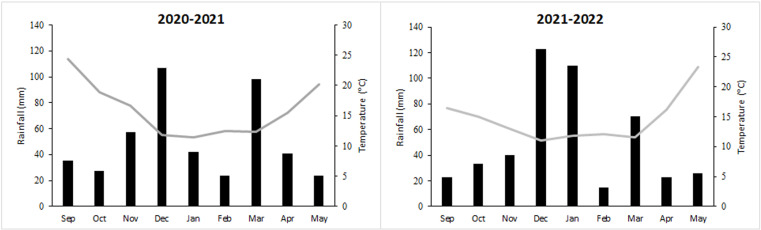
Monthly average temperature (°C) and precipitation (mm) during the two cropping seasons of 2020-2021 and 2021-2022 in Oued Beja Research Station (Oued Beja, Tunisia).

The field trial experiment was conducted only under *O. foetida* infested conditions at Oued Beja. Low to medium *O. foetida* infestation level occurred during the two cropping seasons 2020–2021 and 2021–2022, due to climatic conditions (Fig 4). Thus, untreated plants showed low infestation level with averages 1.81 and 4.49 g of *O. foetida* emerged spikes and DW per plant, respectively.

Field evaluation results showed significant differences between the different tested treatments. Seed coating have boosted the faba bean resistance to *O. foetida* resulting in reduced parasite infestation and improved seed yield (Tables 2 and 3). During the cropping season 2020–2021, BTH, SA1 and SA2 treatments significantly reduced orobanche incidence and enhanced grain yield per plant (162.86, 77.14 and 60.00%, respectively). Severity was significantly reduced after BTH and Trianum treatments. However, no significant effect on orobanche number and 100 seed weight was observed in response to different seed coating treatments. During the cropping season 2021–2022, Panoramix, SA2 and BTH significantly decreased the number of *O. foetida* shoots per plant with 34.06, 32.97 and 26.37%, respectively. Both SA2, Trianum and BTH treatments significantly enhanced seed yield per plant (388.46, 138.46 and 92.31%, respectively). Over the two cropping seasons, SA1 and SA2 treatments have significantly decreased the orobanche number per plant. Results showed also significant increases of 196.77% and 129.03% in grain yield per plant in response to SA2 and BTH treatments, respectively. No significant differences in Incidence and Severity were observed between the different seed priming/coating and control treatments.

**Table 2:** Effects of chemical and biological tolerance inducers on *O. foetida* infestation levels in faba bean assessed in high *O. foetida* infested field.

	Incidence (%)			Severity			Orobanche number/ plant		
	20–21	21–22	Mean	20–21	21–22	Mean	20–21	21–22	Mean
Control	96.67 b	95.00 a	95.83 ab	8.00 b	7.75 ab	7.88 ab	2.36 a	1.82 b	2.09 b
SA1	80.00 a	96.67 a	88.33 ab	7.67 ab	8.25 ab	7.96 ab	1.68 a	1.45 ab	1.56 a
SA2	80.00 a	93.33 a	86.67 a	7.33 ab	6.50 a	6.92 a	1.96 a	1.22 a	1.59 a
BTH	83.33 a	100.00 a	91.67 ab	6.67 a	8.33 ab	7.50 ab	2.13 a	1.34 a	1.73 b
Trianum	90.00 ab	93.33 a	91.67 ab	6.67 a	8.33 ab	7.50 ab	1.86 a	1.44 ab	1.65 b
Serenade	96.67 b	100.00 a	98.33 b	7.67 ab	8.67 b	8.17 b	2.53 a	1.42 ab	1.98 b
Panoramix	96.67 b	91.67 a	94.17 ab	7.67 ab	8.33 ab	8.00 ab	2.42 a	1.20 a	1.81 b

Data with the same letter in the column are not significantly different (Duncan, P = 0.05).

**Table 3:** Effects of chemical and biological tolerance inducers on seed yield and 100 seed weight *O. foetida* infestation in faba bean assessed in high infested *O. foetida* field.

	Seed yield (g/plant)			100 seed weight (g)		
	20–21	21–22	Mean	20–21	21–22	Mean
Control	0.35 a	0.26 ab	0.31 ab	38.53 a	48.00 a	43.27 a
SA1	0.62 b	0.32 ac	0.47 bc	34.20 a	31.31 a	32.75 a
SA2	0.56 b	1.27 d	0.92 d	34.72 a	45.13 a	39.93 a
BTH	0.92 c	0.50 c	0.71 cd	40.91 a	32.14 a	36.53 a
Trianum	0.44 ab	0.62 c	0.53 bc	33.21 a	37.45 a	35.33 a
Serenade	0.24 a	0.34 ac	0.29 ab	36.80 a	32.90 a	34.85 a
Panoramix	0.22 a	0.04 a	0.13 a	42.68 a	33.08 a	32.88 a

Data with the same letter in the column are not significantly different (Duncan, P = 0.05).

As mentioned in pot trial, SA and BTH treatments showed the highest reduction in Orobanche infestation compared to untreated plants resulting in a high significant increase in grain yield. However, Panoramix, Trianum-P and Serenade showed a poorer performance for the grain yield and infestation parameters. The superiority of SA and BTH was clearly visible within the different treatments.

## Discussion

This study describes the effect of chemical and biological tolerance inducers on improving faba bean tolerance to *Orobanche* spp. under field and controlled conditions. The choice of product concentrations was based on the literature and their effectiveness in inducing plant resistance to various pathogens [19,33,34,50–52]. The use of different growing conditions in the field, in pots and in square plastic boxes made it possible to obtain additional information in order to better understand the parasitic process and the effects of the different treatments.

Results of the plastic dish experiment showed that, compared to control, all tested treatments have resulted in significant decreases of broomrape seeds germination. BTH treatment showed the highest decreases in Orobanche germination (Fig 1). The number of broomrape attachments was significantly reduced with all treatments. No tubercle necrosis was observed after seed treatments.

In pot experiment, a significant reduction in *O. crenata* number was only observed after SA, BTH and Serenade treatments. However, with *O. foetida*, only SA and BTH seed priming induced reduction in broomrape tubercle number. Similar results were reported in previous studies conducted with *Orobanche* spp. and *Phelipanche* spp. [18,19,33,34,39,53–55]. This decrease in established tubercles could be associated with a significant decrease of orobanche seed germination which might be also related to reduced germination stimulants (strigolactones) production or increased germination inhibitors in host plant root exudates. Other resistance mechanisms acting during orobanche formation might be also involved. In sunflowers, SA seed priming increased the expression levels of pathogenesis-related proteins encoding for chitinase and defensin and the hypersensitive responsive (HR) gene, accompanied by an accumulation of hydrogen peroxide [56]. Several previous studies have reported that chemical tolerance inducers do not have a direct effect on Orobanche seed germination but rather reduce Orobanche attachment to the host root [18,31,36,37,39]. Abbes et al. [19] suggested that the inductive effect of BTH and SA is not associated with herbicidal activity (or toxic effects) but rather with the SAR pathway, which was demonstrated through the foliar applications that provided evidence of the systemic action of induced resistance to broomrape.

The pot experiment results showed that the used chemical and biological tolerance inducers increased shoot dry weight but without significant differences compared to untreated plants. These results are in line with what was reported in previous studies [19,57–59] where the authors mentioned that SA and BTH treatments induced observable growth reduction in host plant species. Heil et al. [60] explained the reduction in biomass as a result of a metabolic competition between biomass production and defense which uses enormous amounts of energy and assimilates. Other studies have reported also that plant seeds’ treatment with elicitors (SA and IAA) resulted in a decrease of Orobanche infestation accompanied by an increase in host biomass production [33,61,62]. In order to increase the effectiveness of broomrape control and improve plant biomass, the treatment dose should be studied and adjusted as suggested by Buschmann et al. [37].

Pot and plastic dishes experiments’ results were confirmed under field conditions. The results from field trials showed that SA and BTH are the most effective in reducing orobanche infestation and in increasing faba bean seed yield. This agrees with studies conducted on faba bean [33] and lentil [34]. Compared to untreated plants, non-significant differences were observed with the other biological tolerance inducers products (Panoramix, Trianum-P and Serenade) under field conditions. Similarly, Peng et al. [46] observed that the biofungicide Serenade (*Bacillus subtilis* QST713) controlled canola clubroot only under controlled conditions, however in open fields this fungicide was not effective.

These three commercial products were tested with several species and pathosystems [46,51,52,63,64]. However, it seems that this is the first time to be used and tested for potential use in broomrape infestation. These products are composed by *Bacillus* spp. (Serenade), *Trichoderma* spp. (Trianum-P), or *Bacillus* spp., *Trichoderma* spp., arbuscular mycorrhizal fungi and polysaccharides (Panoramix). These microorganisms are used independently or in combination with other microorganisms to improve plant development and control abiotic and biotic stress. Thus, *Bacillus* spp. are PGPR widely used as biofertilizers [65] and thus indirectly increase plant growth and yield *via* the induction of systemic resistance [20]. *Trichoderma* spp. are known for their potential role in improving growth, productivity as well as resistance to many biotic and abiotic stresses [44,66–69]. *Trichoderma* spp. are present in several biopesticides, biofertilizers, etc. [43,44]. For AM, several AM fungal species, e.g., *Glomus* spp. and *Rhizophagus* spp. have been used to increase plant performance [22,70,71]. *Bacillus* spp., *Trichoderma* spp. and AM species have been reported by several authors as biological control agents of orobanche spp. [55,72–75].

The defense responses related to these chemical and biological tolerance inducers are often characterized by induction of certain key enzymes of the secondary metabolism such as peroxidase, polyphenol oxidase, phenylalanine ammonialyase and superoxide dismutase leading to increased phenolic compounds production which can play several roles in plant defense against pathogens, via the formation of structural barriers and activation of plant defense genes, affecting the vascular connection, making it difficult for the broomrape to attach to the roots of the host plant [29,33,76].

The non-significant effect of the used biological tolerance inducers, especially in field conditions can be explained by a number of factors that need to be taken into consideration, such as the host plant, the parasitic plant and the application method and dose of elicitor [12]. However, the efficiency of the application of these elicitors should be tested again in the future with taking into consideration these factors. In this context, Ayed et al. [64] mentioned the beneficial effect of Panoramix in improving germination, physiological parameters, growth and yield production of durum wheat. These authors indicated that combining seed coating and foliar spray was found more effective than the individual applications, and these results are in concordance with those of Norrie and Keathley [77], Gajc-Wolskaet al. [78] and Sharma et al. [79], who depicted that seed priming accompanied to spray application boost growth and yield in different species. According to Briache et al. [33,34], SA or IAA seed pretreatment combined with foliar application were more efficient in controlling *O. crenata* infestation than only foliar spray.

Finally, our results reveals that faba bean seeds priming with SA and BTH tolerance inducers are able to induce systemic resistance in faba bean against *O. foetida*, contributing thus to improving faba bean yield in *O. foetida* infested areas and providing an additional advantage to genetic resistance against Orobanche. Combining this strategy with tolerant varieties will provide more tools for Orobanche management in faba beans, thereby avoiding environmental impact and phytotoxicity problems caused by the use of chemical treatments. The biological products used in this study do not induce significant tolerance against *O. foetida* infestation, especially in field conditions. In this respect, treatment methods and doses would be adjusted to obtain potential beneficial effects.
